# Supplementary material for: Flower-shaped 2D crystals grown in curved fluid vesicle membranes
Source: Nat Commun. 2024 Apr 24;15:3442. doi: 10.1038/s41467-024-47844-x (PMC11043355; doi:10.1038/s41467-024-47844-x)
Supplement: Supplementary file 1 — Supplementary Information [file 41467_2024_47844_MOESM1_ESM.pdf]

Supplementary Information for

Flower-shaped 2D crystals grown in curved fluid vesicle membranes

Hao Wan,[1] Geunwoong Jeon,[2] Weiyue Xin,[3] Gregory M. Grason,[1] and Maria M. Santore[1,*]

1. Department of Polymer Science and Engineering, University of Massachusetts, 120 Governors Drive, Amherst, MA 01003, USA
2. Department of Physics, University of Massachusetts, 710 N. Pleasant Street, Amherst, MA 01003, USA
3. Department of Chemical Engineering, University of Massachusetts, 686 N. Pleasant Street, Amherst, MA 01003, USA

Table of contents

Supplementary Figures 2

Supplementary Note 1. Estimating solid area fraction from the phase diagram 9

Supplementary Note 2. Solid area fraction calculation from vesicle images 11

Supplementary Note 3. Surface evolver model of fluid-solid composite vesicles 23

Supplementary References 28

Supplementary Figures

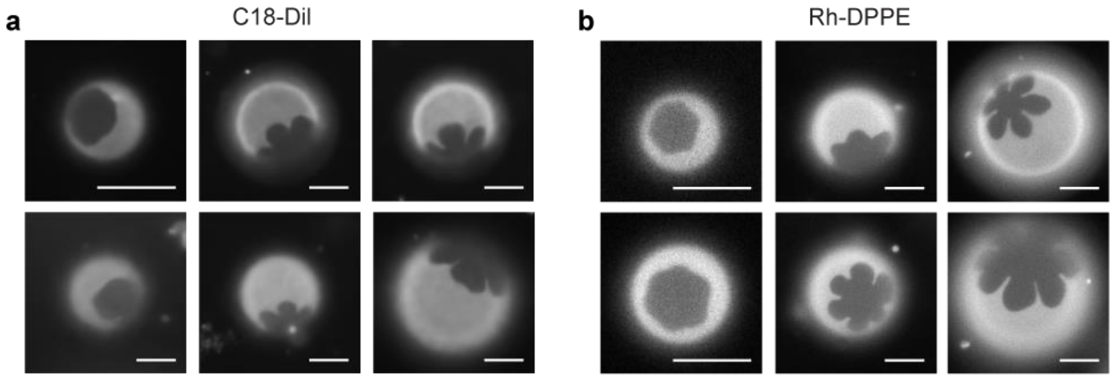

Supplementary Fig. 1. Vesicles containing other tracer lipids. a C18-DiI or **b** Rh-DPPE are employed as tracers instead of the Rh-DOPE employed in the main paper. Vesicles and processing history are otherwise identical to those in the main paper. Scale bars are 10 μm.

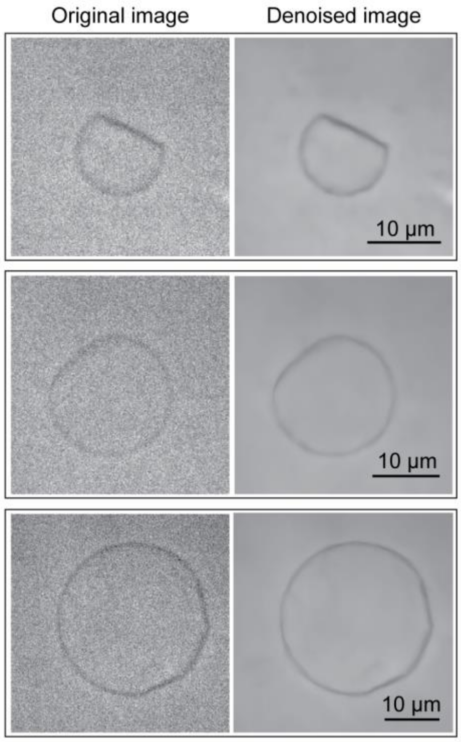

Supplementary Fig. 2. Raw images corresponding to the bright field images of Figure 3.

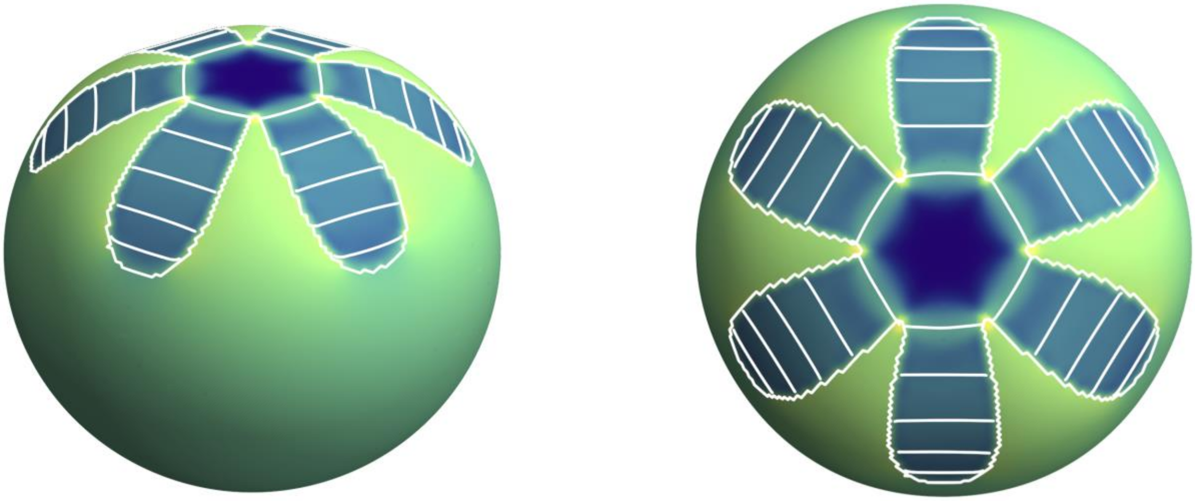

Supplementary Fig. 3. Simulated vesicle shape for $\alpha = 3.5$ with mean curvature distribution mapped as in main text Figure 4. Solid white lines on the "petals" of the solid domain are computationally-derived generators of bending, computed from the streamlines of the flat principal curvature director on the solid domain.

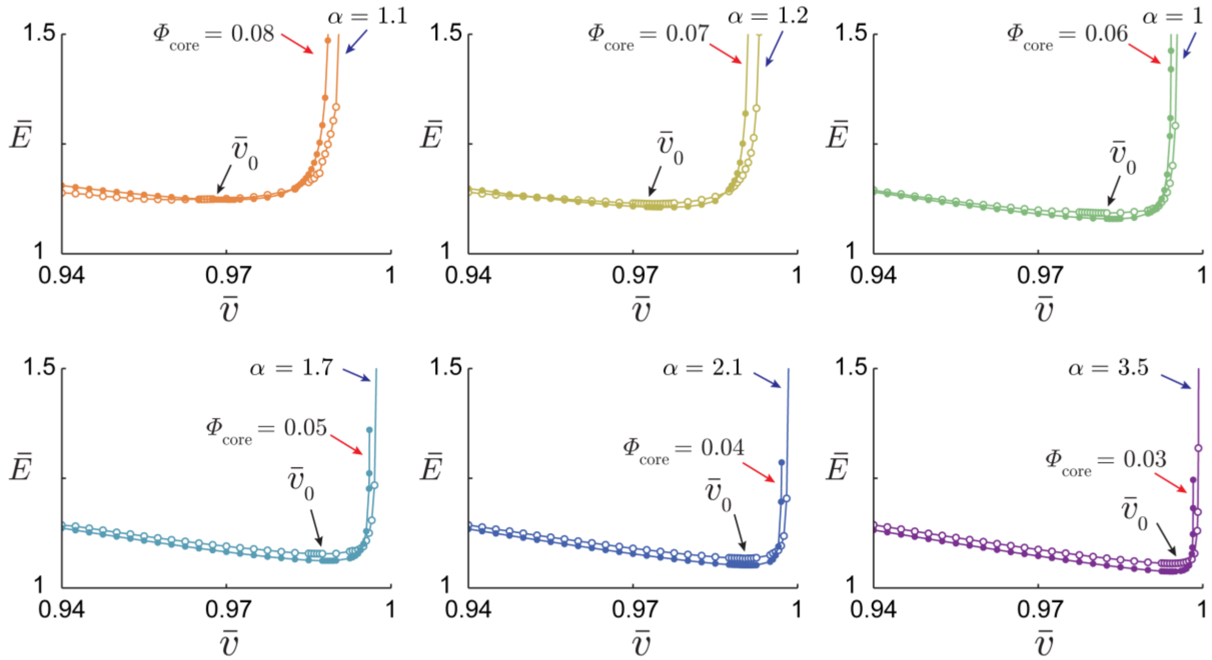

Supplementary Fig. 4. Comparison of elastic energy vs. inflation for 14% solid/variable petal size domains to "effective core" models in Figure 4. For each value of α, the area fraction of a rigid core $\Phi_{\rm core}$ is chosen to match the minimal energy value of reduced volume, $\bar{v}_0$. Notably, the elastic energy of flower shaped domains and simpler effective core model match well even far from this vanishing tension point, particularly in the diverging-energy, high-tension regime as vesicles approach maximal inflation.

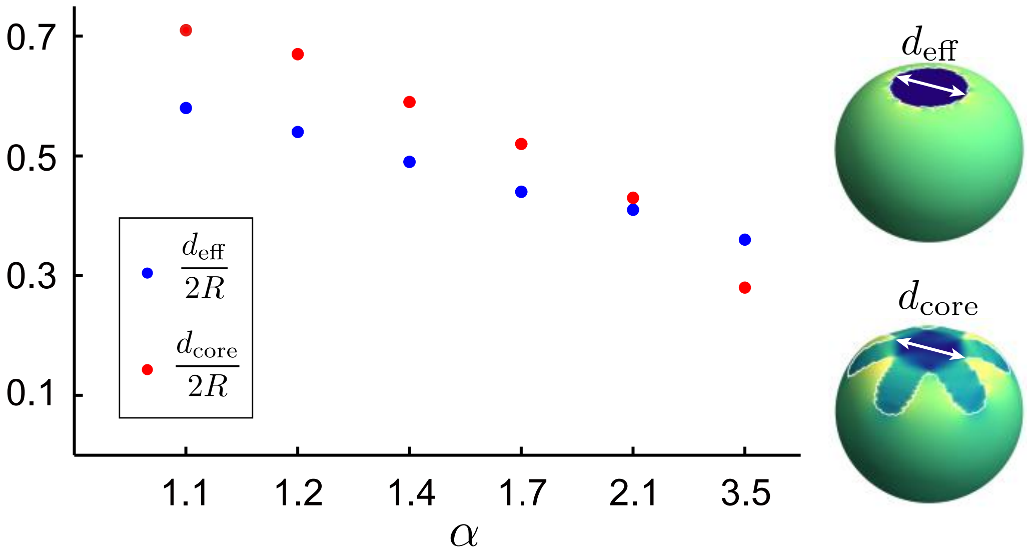

Supplementary Fig. 5. Comparison of the size of the effective core region, d_{eff} (blue circle), to the in-radius size of flowered solid shapes, d_{core} (red circle).

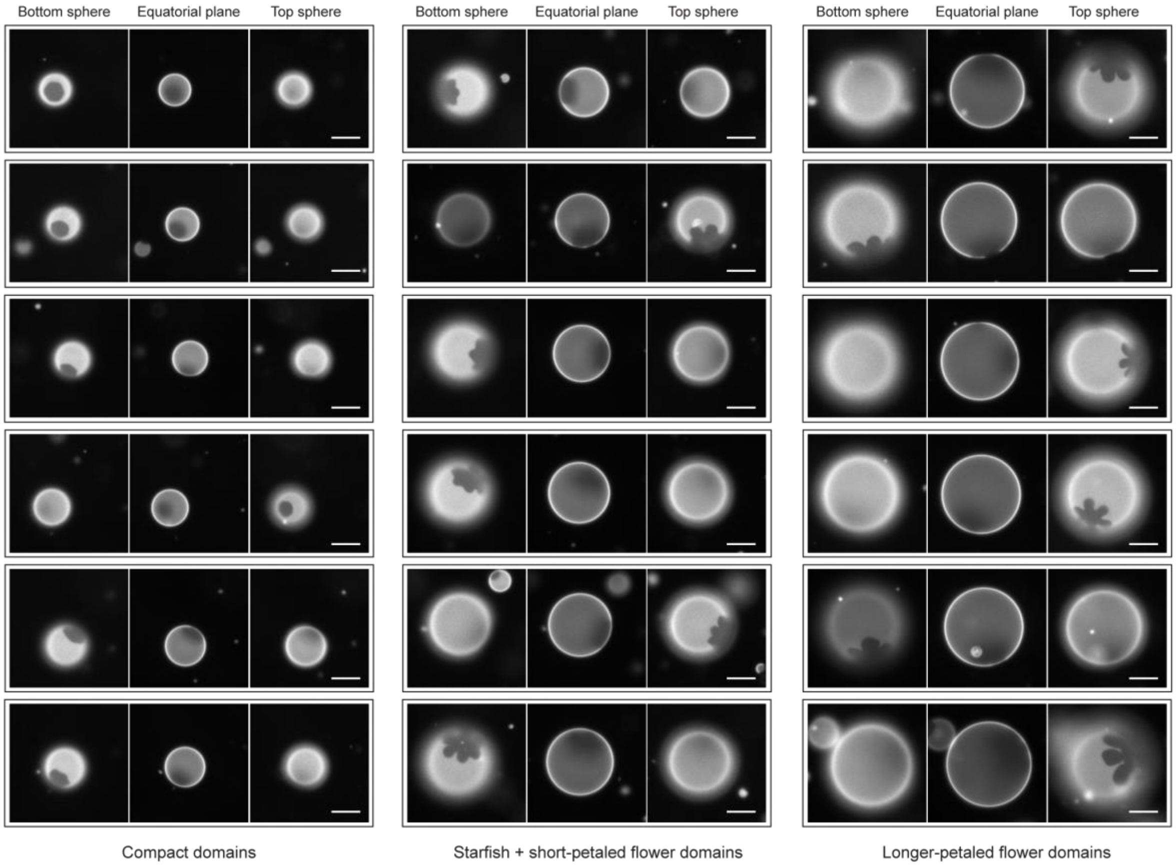

Supplementary Fig. 6. Vesicles containing 20 wt% DPPC/ 80 wt% DOPC. 20% DPPC is used instead of 30% DPPC employed in the main paper. Tracer lipid (Rh-DOPE) and processing history are otherwise identical to those in the main paper. Vesicles are classified into three categories based on solid shapes. For each vesicle, fluorescence micrographs show different focus planes for top, equatorial, and bottom views. Scale bars are 10 μm.

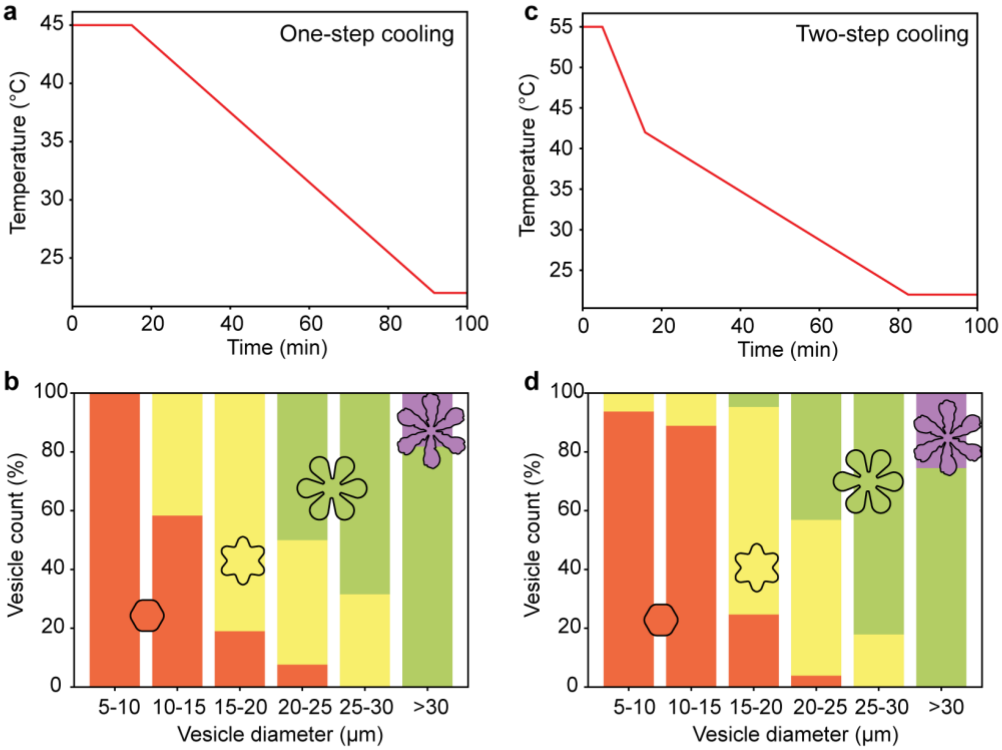

Supplementary Fig. 7. Comparison of one-step cooling (a, b) and two-step cooling (c, d). Thermal history (**a**) and distribution of crystal shapes on vesicles of different sizes (**b**) for a one-step cooling history of 0.3 °C min^{-1}. Thermal history (**c**) and distribution of crystal shapes on vesicles of different sizes (**d**) for a two-step cooling. The one-step cooling data in (**b**), includes 151 vesicles in a single run. The two step cooling data in (**d**), also shown in main text Fig. 2c, include 330 vesicles from 3 runs.

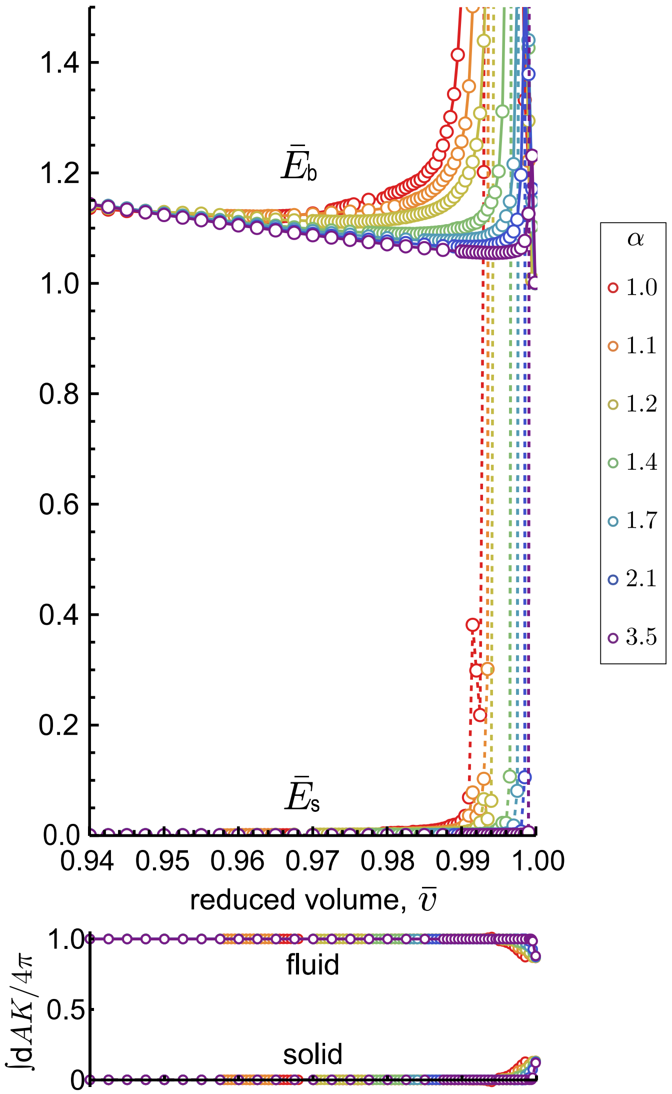

Supplementary Fig. 8. Reduced bending ($\bar{E}_b$), solid strain energy ($\bar{E}_s$) and fluid/solid Gaussian curvature (*K*) distribution of Surface Evolver calculations of fluid-solid composite vesicles, shown in main text Figure 4b. Notably, solid strain energy is negligible relative to bending elastic energy over the experimentally relevant range of energetics (i.e., $\bar{E} \sim 1 - 10$) while the lower plot shows that the Gaussian curvature is localized to the fluid phase in this same regime.

Supplementary Note 1. Estimating solid area fraction from the phase diagram

Here, we use simple mass balance (lever arm rule) to estimate the solid area fraction from the phase diagram for the two-component DOPC/DPPC lipid vesicle membrane. A detailed derivation of the solid area fraction is found in the supporting information of a work by Chen and Santore.[1]

The solid area fraction ϕ, of the vesicle surface containing solid domains, at equilibrium, is

$$\phi = \left(1 + \frac{1}{c}\frac{z_\mathrm{A} - y_\mathrm{A}}{x_\mathrm{A} - z_\mathrm{A}}\right)^{-1} \tag{1.1}$$

Here $c \equiv \underline{A}_\mathrm{S}/\underline{A}_\mathrm{L}$ is the ratio of the lipid molar area in the solid phase, $\underline{A}_\mathrm{S}$, to that in the fluid phase, $\underline{A}_\mathrm{L}$. c depends on the particular solid and fluid and is temperature dependent.

For a phase-separated vesicle at room temperature, x_A, y_A are the DPPC mole fractions in the fluid and solid phases, respectively. z_A is the overall DPPC mole fraction.

The molecular lipid area in the fluid phase is estimated as the molecular area of DOPC in the fluid phase since DOPC is the fluid phase component ($\underline{A}_\mathrm{L} \approx A^\mathrm{F}_\mathrm{DOPC}(@\ 22\ ℃)$). Nagle and Tristram-Nagle[2] report the area per lipid molecule for DOPC in the fluid L_α phase as $A^\mathrm{F}_\mathrm{DOPC}(@\ 30\ ℃) = 72.5\ \text{Å}^2$. The area for DOPC at room temperature is then adjusted using the area thermal expansivity $\kappa = (1/A)(\partial A/\partial T)_\tau$, with a value of 0.003 $℃^{-1}$ based on similar treatment from Nagle and Tristram-Nagel.[2] This gives $A^\mathrm{F}_\mathrm{DOPC}(@\ 22\ ℃) = A^\mathrm{F}_\mathrm{DOPC}(@\ 30\ ℃)\ e^{\kappa \Delta T} = 70.8\ \text{Å}^2$.

The solid phase molecular lipid area is estimated as the molecular area of DPPC in the ripple $\mathrm{P}_{\beta'}$ phase at 22 ℃ ($\underline{A}_\mathrm{s} \approx A^\mathrm{S}_\mathrm{DPPC}(@\ 22\ ℃)$) since solid domains have nearly pure DPPC content. Nagle and Tristram-Nagle[2] report the area per DPPC molecule in the fluid phase as

$A^{\mathrm{F}}_{\mathrm{DPPC}}(@\ 50\ ℃) = 64.0\ Å^2$. The extrapolated molecular area for DPPC at 22 ℃ ($A^{\mathrm{S}}_{\mathrm{DPPC}}(@\ 22\ ℃)$) is calculated using κ = 0.003 - 0.006 $℃^{-1}$ from 50 to 42 ℃ for fluid L_α phase, κ = 0.003 - 0.006 $℃^{-1}$ from 41 to 22 ℃ for rippled $P_{\beta'}$ solid phase, and an approximately total 17% areal reduction from the fluid L_α to the rippled $P_{\beta'}$ solid phase through the main transition at 41- 42 ℃.[3] This gives $A^{S}_{\mathrm{DPPC}}(@\ 22\ ℃)$= 45.2 - 49.0 $Å^2$.

According to the phase diagram, at room temperature (22 ℃), x_{A} is 0.17 ± 0.02 and y_{A} is approximated as 0.95 since phase separation in DOPC/DPPC mixtures is known to produce solid domains that are nearly pure in DPPC.[4, 5, 6, 7, 8] z_{A} is 0.314 (30 wt% DPPC).

Then, from Equation (1.1), solid area fraction ϕ is estimated to be 11 - 15%. The lower and upper limit are estimated using $A^{\mathrm{S}}_{\mathrm{DPPC}}(@\ 22\ ℃)$ value of 45.2 $Å^2$ and 49.0 $Å^2$, respectively.

If the solid phase contains pure DPPC (y_{A} =1), from Equation (1.1), solid area fraction ϕ is estimated to be 10 - 15%.

Supplementary Note 2. Solid area fraction calculation from vesicle images

2A. Solid area fraction for hexagonal domains

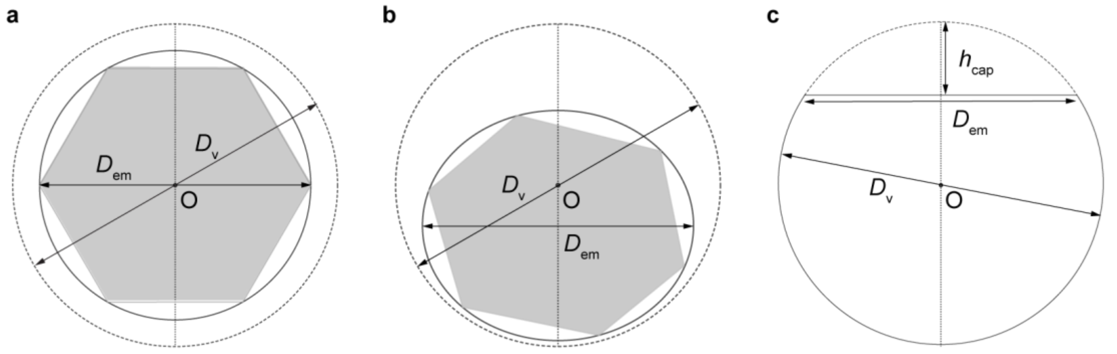

Supplementary Fig. 9. Projections of a hexagonal shape on the flat plane of a truncated sphere, viewed from three different angles. a Perpendicular view demonstrating the hexagon in its original shape. **b** An oblique view resulting in a distorted representation of the hexagon, which resembles a typical micrograph from fluorescent microscopy. **c** Side view

To simplify the estimation of the solid area fraction of a hexagonal domain, the solid hexagonal domain and the fluid part inside the hexagon's circumscribed circle is treated as a plane while the other part of the fluid vesicle is treated as a section of a sphere, as shown in Supplementary Fig. 9c. To calculate the solid area fraction of a hexagonal domain, the following quantities are measured, including:

D_{v}: Vesicle Diameter

D_{em}: Length of the major axis of the ellipse that goes across all the vertices of the deformed hexagon (as shown in Supplementary Fig. 9b). This diameter is irrelevant to the position of the hexagon on the vesicle, as shown in Supplementary Fig. 9a and 9b.

The solid area fraction of the hexagon ϕ_{hex} is then calculated as:

$$\phi_{\mathrm{hex}} = \frac{3\sqrt{2}}{2\pi} \times \frac{\pi\left(\frac{D_{\mathrm{em}}}{2}\right)^2}{4\pi\left(\frac{D_{\mathrm{v}}}{2}\right)^2 - 2\pi\left(\frac{D_{\mathrm{v}}}{2}\right)h_{\mathrm{cap}} + \pi\left(\frac{D_{\mathrm{em}}}{2}\right)^2} \qquad (2.1)$$

where

$$h_{\mathrm{cap}} = \frac{D_{\mathrm{v}}}{2} - \sqrt{\left(\frac{D_{\mathrm{v}}}{2}\right)^2 - \left(\frac{D_{\mathrm{em}}}{2}\right)^2} \quad (2.2)$$

2B. Solid area fraction for flower-shaped domains

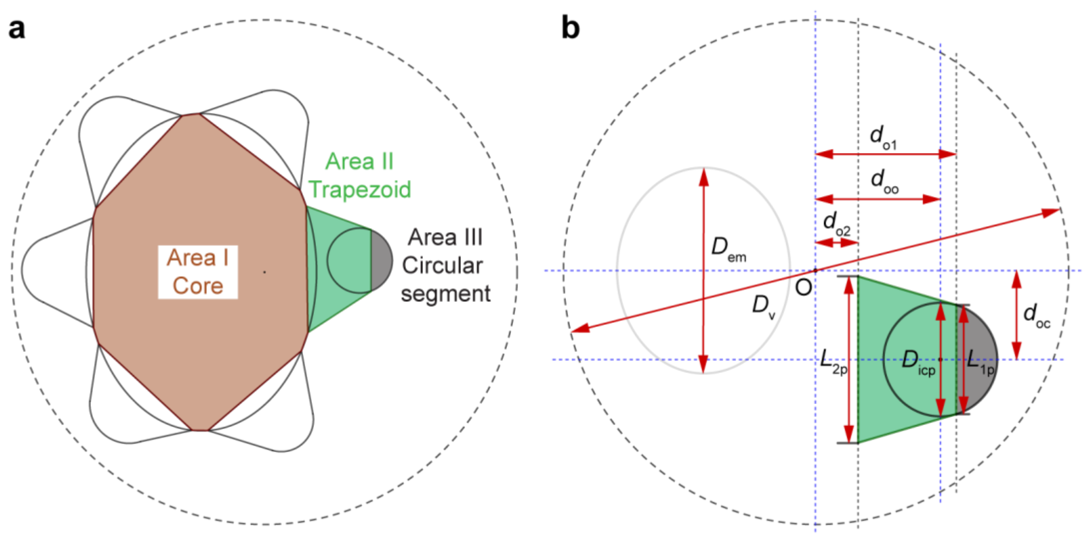

Supplementary Fig. 10. Decomposition of solid fraction estimation for flower-shaped domains. **a** Projection of a ninja star flower shape on a truncated sphere, with a hexagonal core on the flat plane and the petals taking cylindrical curvature, viewed from an oblique angle, which resembles a typical micrograph from fluorescent microscopy. The colored regions (brown for the core, green for the trapezoid, gray for the circular segment) show the decomposition of the flower shape to facilitate solid area calculation. **b** Scheme of all the quantities measured for calculating the solid area fraction of a flower domain. The positions and sizes of the core and petal are altered to facilitate the view of those quantities and may not resemble a real micrograph.

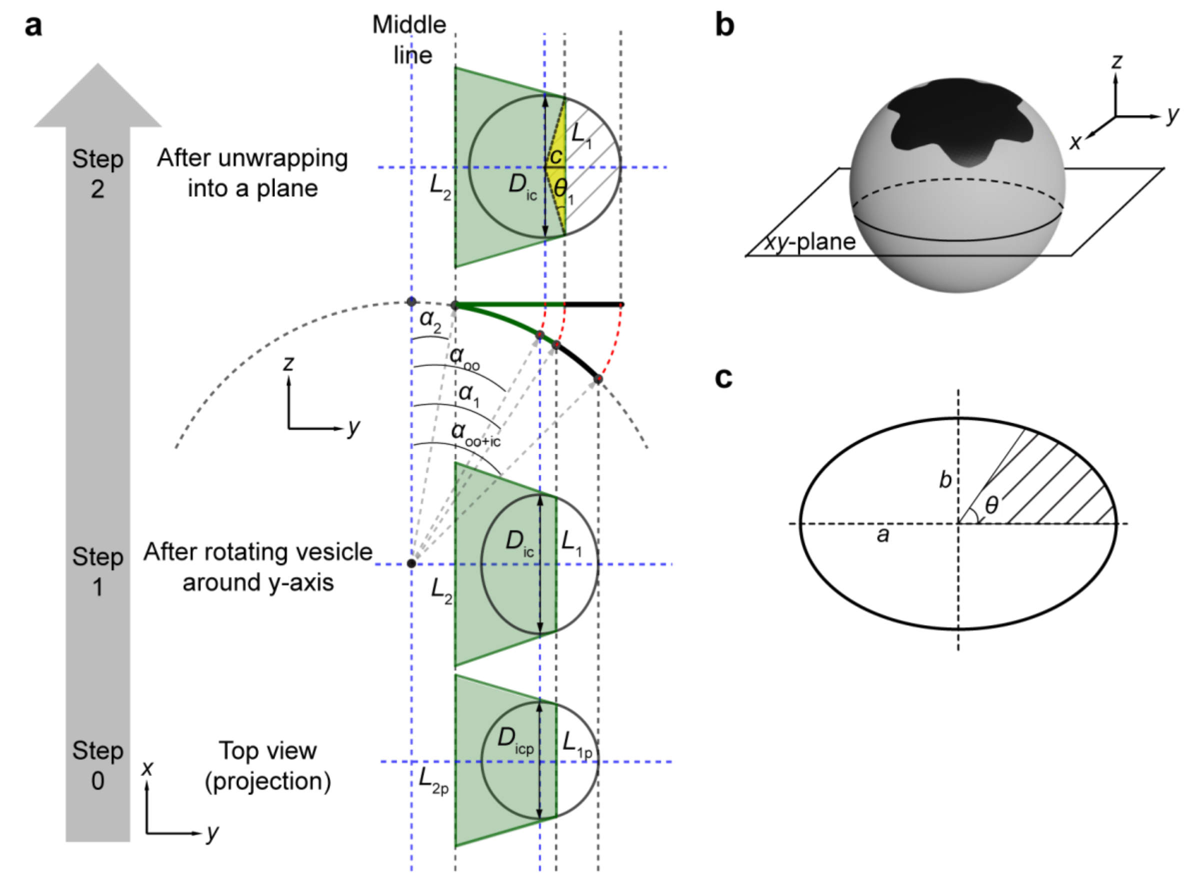

Supplementary Fig. 11. Illustration for the area calculation of a flower petal. a Procedures for transforming the measured quantities on a sphere in the projection view to a planar view. **b** Scheme for a perspective of a vesicle under fluorescent microscopy. A micrograph is taken by moving along the z-axis until a preferred focus plane is chosen. Vesicles with solid domains facing the z-direction are chosen to facilitate area measurement. **c** Scheme for a section (the shaded region) of an ellipse. a, b and θ are the major axis, minor axis, and the polar angle of the ellipse.

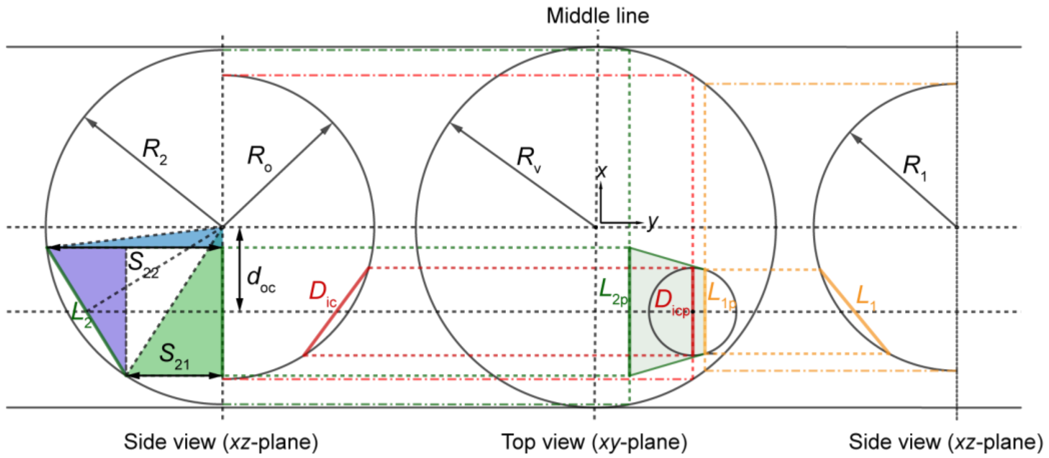

Supplementary Fig. 12. Scheme for calculating the real length of the two bases of the trapezoid (green and red lines) and the diameter of the inscribed circle (yellow lines). The projection length(*xy*-plane) for those quantities corresponds to the chord lengths in corresponding *xz*-planes of the sphere.

To simplify the measurement of the solid area fraction of a flower domain (we use ninja star flower as an example here), the flower domain is assumed to have six-fold symmetry and be composed of a hexagon core (Area I) which is flat and six petals which have cylindrical curvature. The other fluid part of the vesicle is assumed to have identical spherical curvature, ignoring the complexity of the curvature for the connection region between the solid petals contour and the fluid membrane. For the purpose of measuring solid area fraction, the error for this simplified treatment should be small and not enough to influence the result. Each petal is treated as the combination of a trapezoid (Area II) and a section of a circle (Area III) whose center is on the bisector of the trapezoid and the circle is tangent to both vertices of the short base of the trapezoid (Supplementary Fig. 10a). The circle will be named the “inscribed circle” of the trapezoid from now on for simplification.

To measure the solid area fraction of a flower domain, the following quantities (9 in total) are measured, as shown in Supplementary Fig. 10b (the position of the petal and the core in Supplementary Fig. 10b is just for a clear illustration of the quantities and might be different from a real measurement):

D_{v}: Vesicle Diameter

D_{em}: Length of the major axis of the ellipse that go across all the six grooves between the petals.

D_{icp}: Projection length of the diameter of the inscribed circle of the trapezoid.

$L_{1\mathrm{p}}$: Projection length of the short base of the trapezoid

$L_{2\mathrm{p}}$: Projection length of the long base of the trapezoid

d_{o1}: Perpendicular distance between the center of the equatorial circle (Point O) and the line of $L_{1\mathrm{p}}$

d_{o2}: Perpendicular distance of point O to the line of $L_{2\mathrm{p}}$

d_{oo}: Perpendicular distance of point O to the bisector of the inscribed circle of the trapezoid that is parallel to the trapezoid base.

d_{oc}: Perpendicular distance of point O to the bisector of the trapezoid

Then, area I, II and III are calculated as followed:

Area I:

The hexagonal shape in Area I is treated roughly as the inscribed hexagon of the ellipse, although it might not be exactly a perfect hexagon.

$$A_{\mathrm{I}} = \frac{3\sqrt{2}}{2\pi} \times \pi \left(\frac{D_{\mathrm{em}}}{2}\right)^2 \tag{2.3}$$

Area II and Area III:

Before showing the equations for calculating Area II and III, we will first introduce the basic idea of the calculation here. The long base of the trapezoid is connected to the side of the hexagonal shape of Area I. To measure the real area of the petal unwrapped in a plane, we can consider in two steps, as shown in Supplementary Fig. 11a. Since the petal is assumed to have cylindrical curvature, the two bases of the trapezoid are straight lines. A fluorescent image in our experiment is a projection view image in the *xy*-plane from a certain in-focus depth in the z-direction (as shown

in Supplementary Fig. 11b). We can first imagine rotating the vesicle around y-axis to position the petal to the center of the view (Supplementary Fig. 11a Step 0 to Step 1). The real length of the two bases (L_1 and L_2) are chord lengths in corresponding *xz*-plane slice of the vesicle, as shown in Supplementary Fig. 12. Next, we can imagine unwrapping the petal onto a plane, as shown in Supplementary Fig. 11a Step 1 to Step 2, which will elongate features in the bisector direction of the trapezoid. We assume the inscribed circle has an ellipse shape after unwrapping (not sure about the major and minor axis).

In the first step, the length of the two trapezoid bases L_1, L_2 and the diameter of the inscribed circle D_{ic} (now is an ellipse rather than a circle) are calculated. Here, the calculation of L_2 is used as an example (see Supplementary Fig. 12 green lines and Equation (2.4) - (2.7)) and the calculation of L_1 and D_{ic} are similar and omitted here.

$$d_{\mathrm{o2}}{}^2 + R_2{}^2 = \left(\frac{D_{\mathrm{v}}}{2}\right)^2 \tag{2.4}$$

$$S_{21}{}^2 + \left(\frac{L_{\mathrm{2p}}}{2} + d_{\mathrm{oc}}\right)^2 = R_2{}^2 \text{ (Green Triangle)} \tag{2.5}$$

$$S_{22}{}^2 + \left(\frac{L_2'}{2} - d_{\mathrm{oc}}\right)^2 = R_2{}^2 \text{ (Blue Triangle)} \tag{2.6}$$

$$L_{\mathrm{2p}}{}^2 + (S_{22} - S_{21})^2 = L_2{}^2 \text{ (Purple Triangle)} \tag{2.7}$$

Next, the height of the trapezoid (H_{tpz}) is calculated as

$$H_{\mathrm{tpz}} = \begin{cases} R_{\mathrm{v}}(\alpha_1 - \alpha_2), & \text{if trapezoid on one side of the middle line in Supplementary Fig. 11a} \\ R_{\mathrm{v}}(\alpha_1 + \alpha_2), & \text{if the trapezoid goes across the middle line, not shown here} \end{cases} \tag{2.8}$$

$$\alpha_1 = \arcsin\frac{d_{\mathrm{o1}}}{R_{\mathrm{v}}} \tag{2.9}$$

$$\alpha_2 = \arcsin\frac{d_{\mathrm{o2}}}{R_{\mathrm{v}}} \tag{2.10}$$

Then, the area of the trapezoid (Area II) is:

$$A_{\mathrm{II}} = (L_1 + L_2) \times \frac{H_{\mathrm{tpz}}}{2} \tag{2.11}$$

The area of the ellipse segment (Area III) is the difference between the area of unwrapped ellipse section and the small triangle (as shown in Supplementary Fig. 11a). To calculate area III, the following quantities need to be introduced, including the major (a) and minor axis (b) of the unwrapped ellipse, the height of the triangle (c), and the angle θ_1, as shown in Supplementary Fig. 11a.

As shown in Supplementary Fig. 11c, for a section of an ellipse, the area is

$$A_{\text{elp section}} = \frac{ab}{2} \times \tan^{-1}\left(\frac{a}{b}\tan\theta\right) \tag{2.12}$$

For the case in Supplementary Fig. 11a, R_{ic} ($D_{\text{ic}}/2$) is either the major or minor axis, and R'_{ic} is defined as the other axis. R'_{ic} is calculated as followed:

$$R'_{\text{ic}} = R_{\text{v}} \times (\alpha_{\text{oo+ic}} - \alpha_{\text{oo}}) \tag{2.13}$$

$$\alpha_{\text{oo}} = \arcsin\frac{d_{\text{oo}}}{R_{\text{v}}} \tag{2.14}$$

$$\alpha_{\text{oo+ic}} = \arcsin\frac{d_{\text{oo}} + R_{\text{ic}}}{R_{\text{v}}} \tag{2.15}$$

The height of the triangle (c) is:

$$c = R_{\text{v}} \times (\alpha_1 - \alpha_{\text{oo}}) \tag{2.16}$$

The angle θ_1 is:

$$\theta_1 = \tan^{-1}\frac{2c}{L_1} \tag{2.17}$$

After substitute a, b and θ in Equation (2.12) for corresponding quantities for the case in Supplementary Fig. 11a, we get

$$A'_{\text{elp section}} = \begin{cases} R_{\text{ic}}R'_{\text{ic}} \times \tan^{-1}\left(\dfrac{R'_{\text{ic}}}{R_{\text{ic}}}\cot\theta_1\right) \text{ if } R_{\text{ic}} < R'_{\text{ic}} \\ R_{\text{ic}}R'_{\text{ic}} \times \left(\dfrac{\pi}{2} - \tan^{-1}\left(\dfrac{R_{\text{ic}}}{R'_{\text{ic}}}\tan\theta_1\right)\right) \text{ if } R_{\text{ic}} > R'_{\text{ic}} \end{cases} \tag{2.18}$$

The area of the small triangle is:

$$A_{\text{triangle}} = \frac{cL_1}{2} \tag{2.19}$$

Then, Area III is:

$$A_{\mathrm{III}} = A'_{\mathrm{elp\ section}} - A_{\mathrm{triangle}} \tag{2.20}$$

The area of a flower is:

$$A_{\mathrm{flower}} = A_{\mathrm{I}} + 6(A_{\mathrm{II}} + A_{\mathrm{III}}) \tag{2.21}$$

The solid area fraction of the flower domain is:

$$\phi_{\mathrm{flower}} = \frac{A_{\mathrm{flower}}}{4\pi\left(\frac{D_{\mathrm{v}}}{2}\right)^2 - 2\pi\left(\frac{D_{\mathrm{v}}}{2}\right)h_{\mathrm{cap}} + \pi\left(\frac{D_{\mathrm{em}}}{2}\right)^2} \tag{2.22}$$

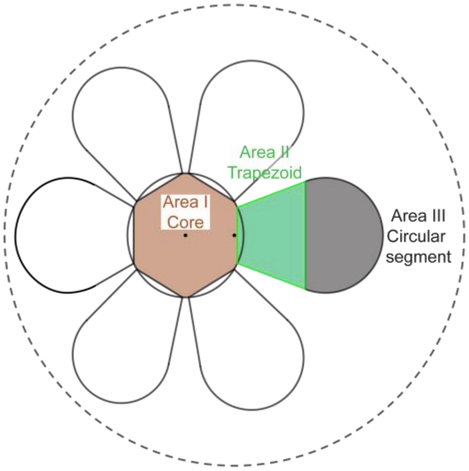

Supplementary Fig. 13. Projection of a simple flower shape on a truncated sphere, with a hexagonal core on the flat plane and the petals taking cylindrical curvature, viewed from an oblique angle, which resembles a typical micrograph from fluorescent microscopy. The colored regions show the decomposition of the flower shape to facilitate solid area calculation, which resembles the case of ninja star flower in Supplementary Fig. 10a.

In the previous case, ninja star flower was used as an example for solid area fraction calculation. For a simple flower as shown in Supplementary Fig. 13, the shapes can be divided to three parts as well, including the hexagonal shape core (Area I), the trapezoid (Area II), and the circular segment (Area III). The difference is that the "inscribed circle" of the trapezoid here is tangent to both vertices of the long base of the trapezoid. As a result, the procedure for calculating the solid

area is almost the same as the previous case, and only minor revisions are needed. Here, only the equations that differ from the previous case are shown and all other equations are the same as before.

$$H_{\mathrm{tpz}} = \begin{cases} R_{\mathrm{v}}|\alpha_1 - \alpha_2|, & \text{if the trapezoid is on one side of the middle line} \\ R_{\mathrm{v}}(\alpha_1 + \alpha_2), & \text{if the trapezoid go across the middle line} \end{cases} \quad (2.8')$$

$$R'_{\mathrm{ic}} = \begin{cases} R_{\mathrm{v}}(\alpha_{\mathrm{ic-oo}} + \alpha_{\mathrm{oo}}), & \text{if O is between center of the inscribed circle and petal edge} \\ R_{\mathrm{v}}(\alpha_{\mathrm{oo+ic}} - \alpha_{\mathrm{oo}}), & \text{for almost all other circumstances} \end{cases} \quad (2.13')$$

Where,

$$\alpha_{\mathrm{ic-oo}} = \frac{\arcsin(R_{\mathrm{ic}} - d_{\mathrm{oo}})}{R_{\mathrm{v}}} \quad (2.15')$$

$$c = R_{\mathrm{v}}|\alpha_2 - \alpha_{\mathrm{oo}}| \quad (2.16')$$

$$\theta_1 = \tan^{-1}\frac{2c}{L_2} \quad (2.17')$$

$$A'_{\mathrm{elp\ section}} = \begin{cases} \pi R'_{\mathrm{ic}} R_{\mathrm{ic}} - R_{\mathrm{ic}} R'_{\mathrm{ic}} \times \tan^{-1}\left(\frac{R'_{\mathrm{ic}}}{R_{\mathrm{ic}}}\cot\theta_1\right) & \text{if } R_{\mathrm{ic}} < R'_{\mathrm{ic}} \\ \frac{\pi}{2} R'_{\mathrm{ic}} R_{\mathrm{ic}} + R_{\mathrm{ic}} R'_{\mathrm{ic}} \times \tan^{-1}\left(\frac{R_{\mathrm{ic}}}{R'_{\mathrm{ic}}}\tan\theta_1\right) & \text{if} R_{\mathrm{ic}} > R'_{\mathrm{ic}} \end{cases} \quad (2.18')$$

$$A_{\mathrm{triangle}} = \frac{cL_2}{2} \quad (2.19')$$

$$A_{\mathrm{III}} = A'_{\mathrm{elp\ section}} + A_{\mathrm{triangle}} \quad (2.20')$$

2C. Images of solid area fraction measurement

Based on the methods mentioned above, ≈ 15 vesicles for each shape were measured for solid area fraction. The original images and their corresponding solid area fraction (ϕ) are shown in Supplementary Fig. 14-16 for hexagonal, ninja star flower, and simple flower domain respectively.

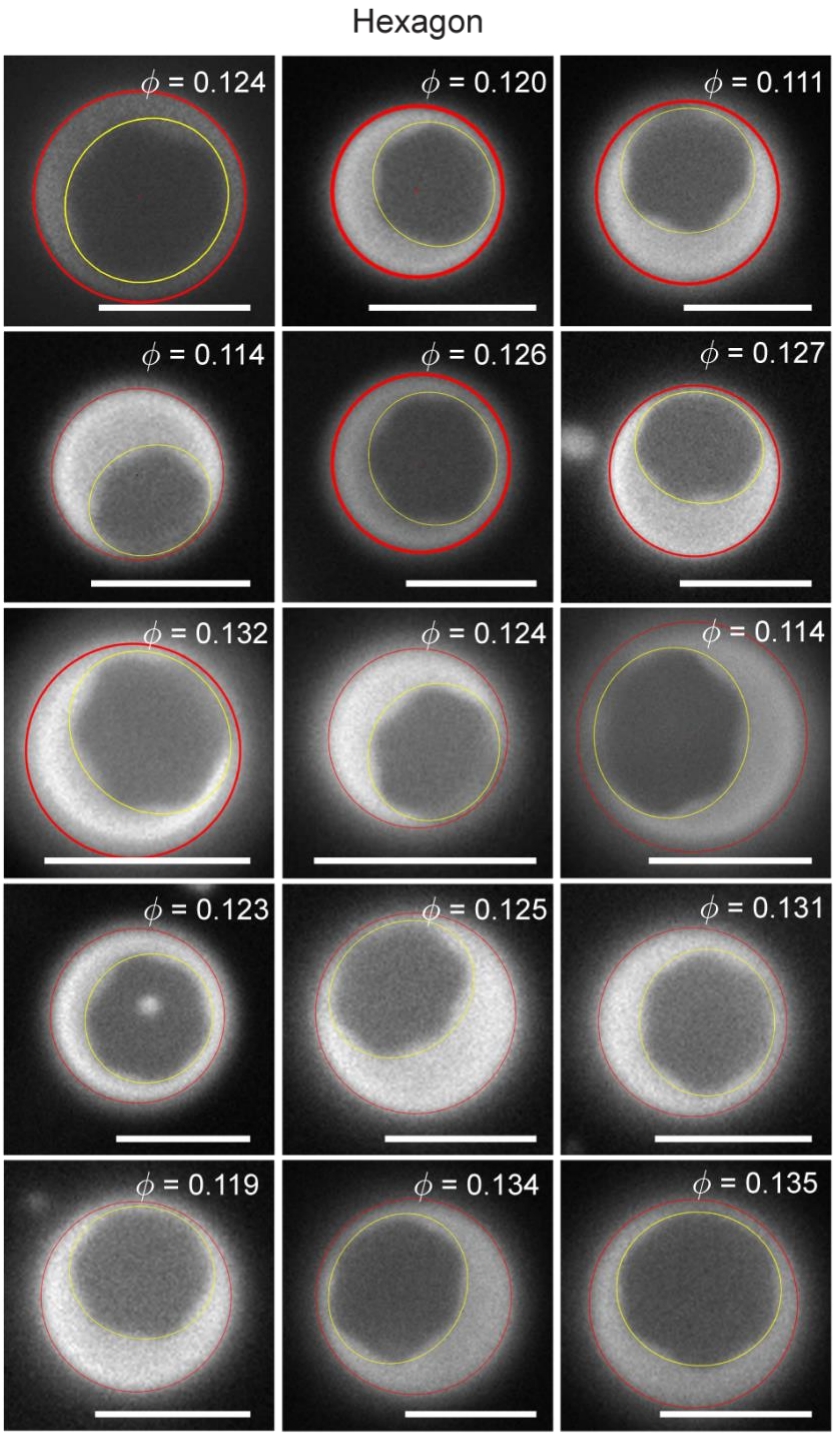

Supplementary Fig. 14. Fluorescent images and corresponding solid area fraction for 15 different vesicles with hexagonal domains. Embedded circles in each micrograph show the measured quantities based on Supplementary Note 2A. Scale bars are 10 μm.

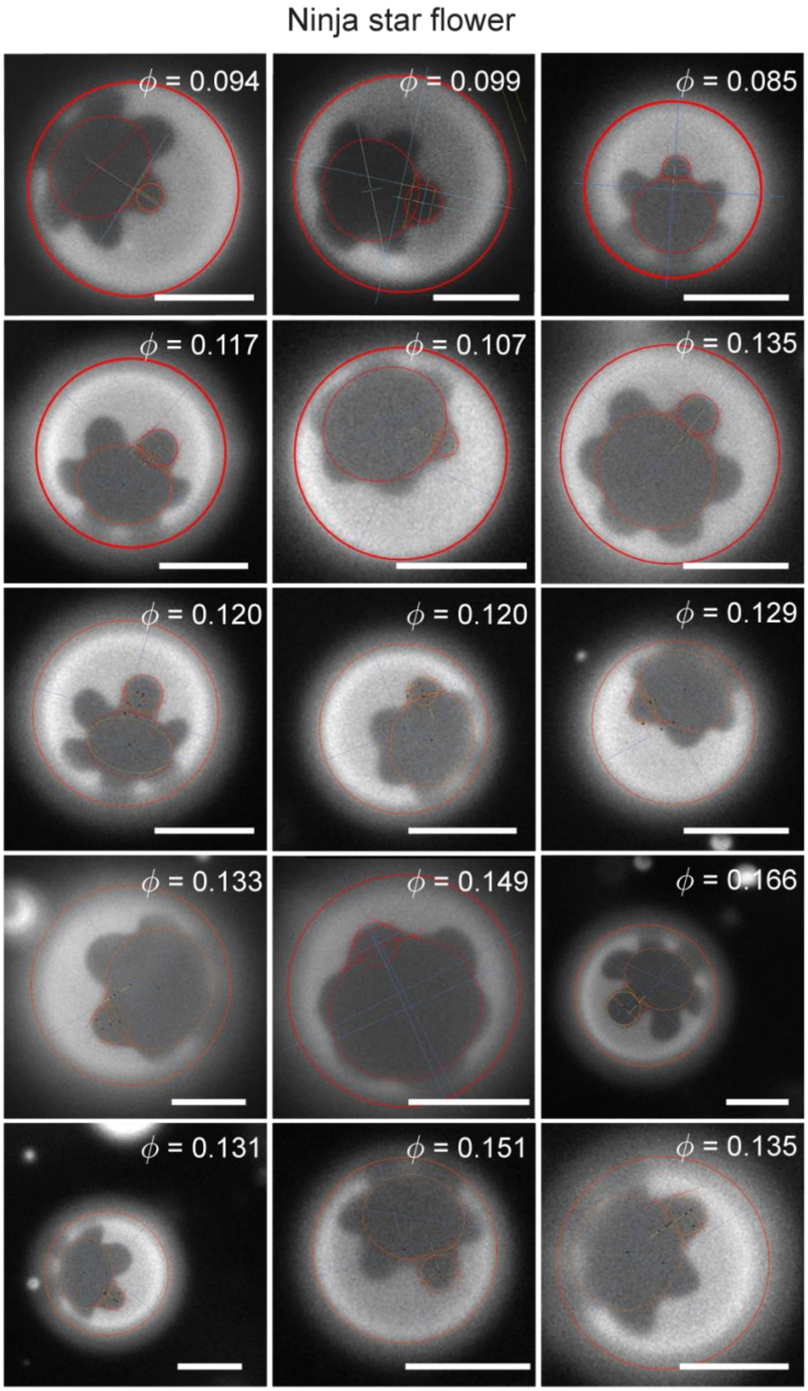

Supplementary Fig. 15. Fluorescent images and corresponding solid area fraction for 15 different vesicles with ninja star flower domains. Embedded circles and lines in each micrograph show the measured quantities based on Supplementary Note 2B. Scale bars are 10 μm.

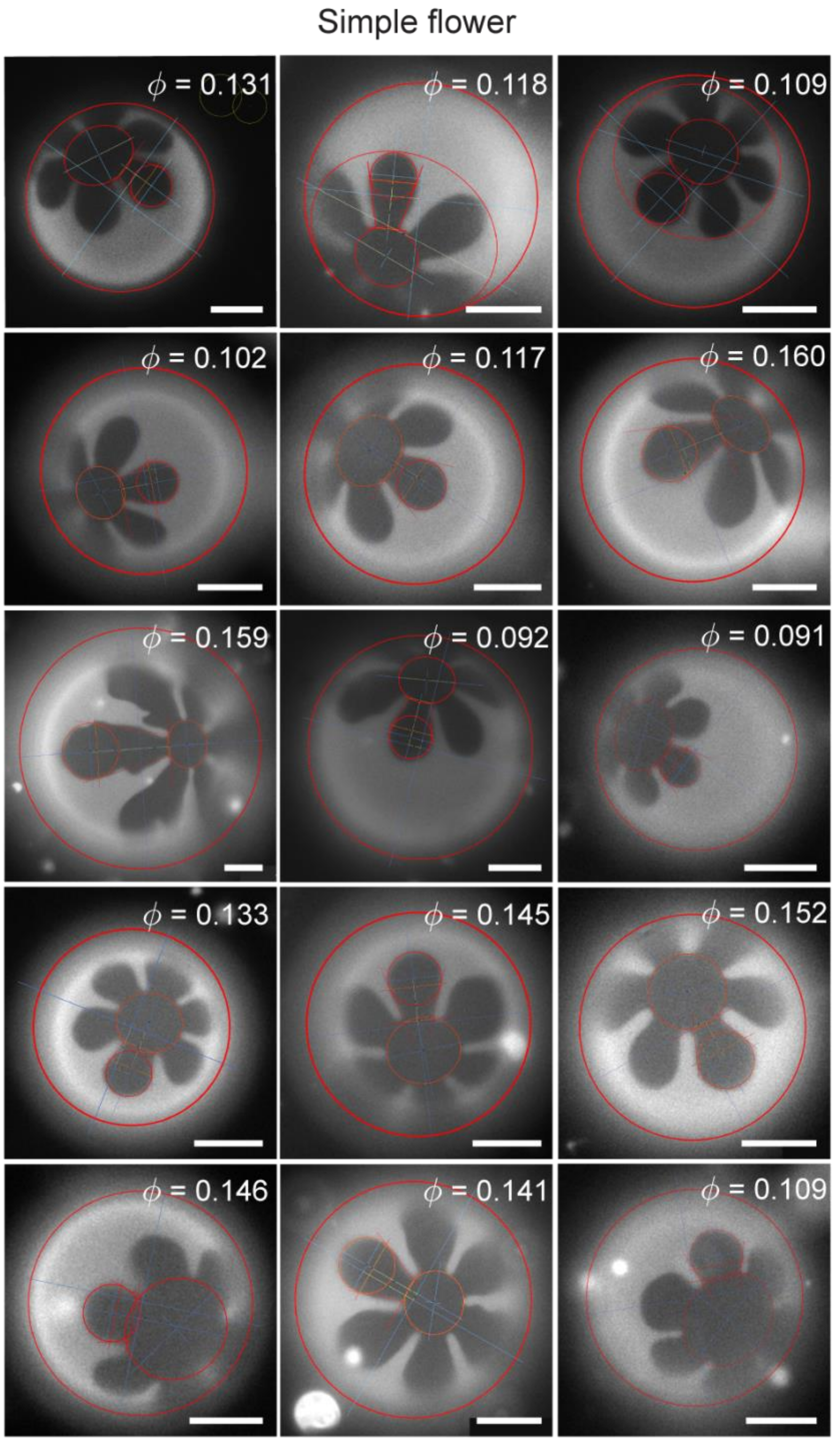

Supplementary Fig. 16. Fluorescent images and corresponding solid area fraction for 15 different vesicles with simple flower domains. Embedded circles and lines in each micrograph show the measured quantities based on Supplementary Note 2B. Scale bars are 10 μm.

Supplementary Note 3. Surface evolver model of fluid-solid composite vesicles

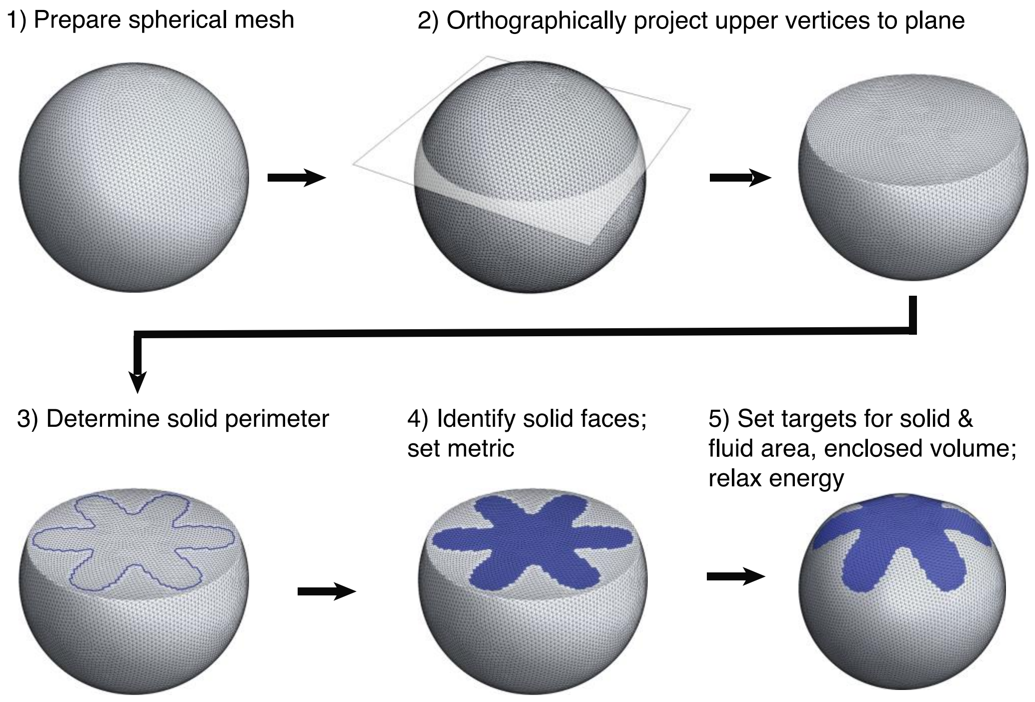

Supplementary Fig. 17. Schematic overview of the workflow for Surface Evolver calculations of composite vesicle shapes and energetics.

Here we describe the procedure to numerically compute optimal elastic energy shapes of composite vesicles. The workflow of our numerical calculation protocol is shown in Supplementary Fig. 17. The elements of each step are summarized as follows:

1) *Initialize spherical mesh* – We initialize a spherical mesh of ~10^5 triangular facets.
2) *Flatten upper plane* – To initialize the solid domain, the vertices above a height z are orthographically projected down to that plane. The height z is chosen to ensure that the solid domain fits on top of the planar cut.

3) *Determine the solid perimeter* – A shape for the boundary edge of the solid domain is selected (with a given α value and shape described below) and projected onto the plane at constant z, centered around the pole of axisymmetry of the "dented sphere".
4) *Prepare solid faces* – Faces interior to the 2D stencil of the domain edge (i.e., with all three vertices interior to the boundary curve) are selected as the solid domain. The data on the facet edge lengths and dot products in this planar state is used to generate the (i.e., flat metric) reference state of the elastic strain energy of the solid (detailed below).
5) *Set targets and relax energy* – Starting from this configuration, the elastic energy of the entire vesicle (detailed below) is relaxed in Surface Evolver, targeting constraints on both solid and fluid areas as well as the internal volume.

For the effective core model, the procedure is similar, with the exception that circular planar domains are held fixed (i.e., strictly rigid) in the subsequent energy relaxation.

2D domain shape - The shape of the solid domain was defined by the following radial function on the planar surface.

$$r(\theta) = r_0 + \frac{1}{2} a r_0 \cos(6\theta) - \frac{1}{10} a r_0 \cos(12\theta) \quad (3.1)$$

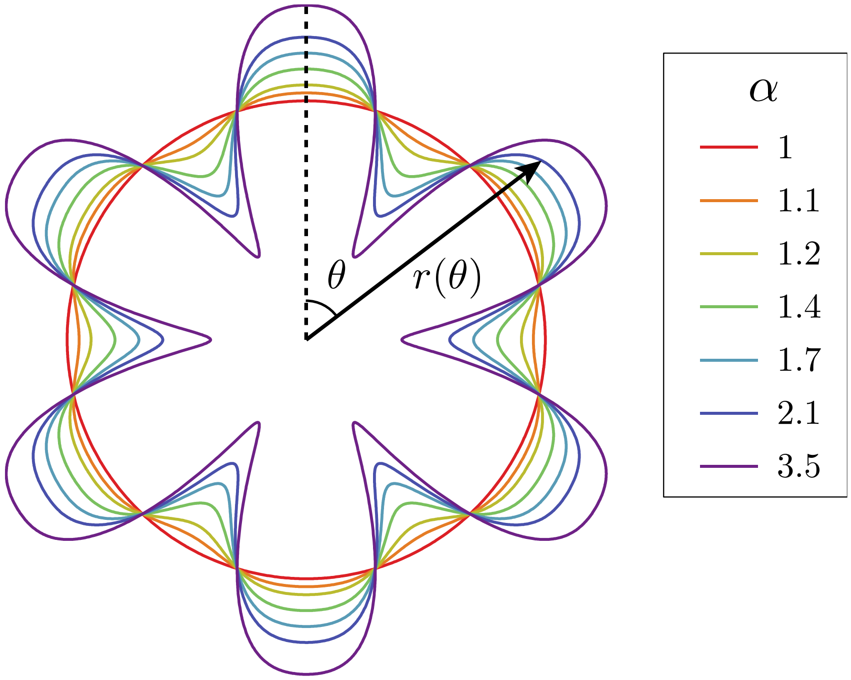

Supplementary Fig. 18. Perimeter shapes for 2D solid domains used in Surface Evolver calculations.

which implies $\alpha = r(0)/r(\pi/6) = (5 + 2a)/(5 - 3a)$. The value of r_0 was chosen for each α to keep the initial area fraction as close as possible to 14% solid fraction, although subsequent relaxation of the fluid area was needed to more exactly match this ratio. The ratio between the first and second harmonic was selected to give closer approximation to the variably-petaled shapes observed in experiments over the full range of α, shown in Supplementary Fig. 18.

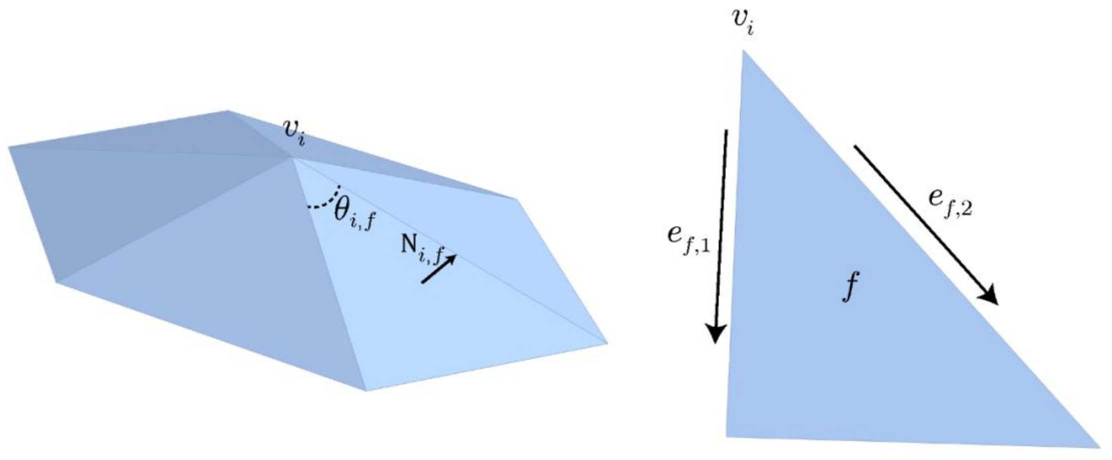

Supplementary Fig. 19. Discrete mesh components around a given central vertex v_i .

Mesh geometry – Based on the mesh geometry defined in Supplementary Fig. 19, we use discrete approximants for mean (h_i) and Gaussian (k_i) curvature at vertex $\mathbf{v}_i$.[9, 10] The discrete mean curvature follows from the gradient of the area element normal to the mesh,

$$h_i = \frac{1}{2}\frac{\mathbf{F}_i \cdot \mathbf{N}_i}{\mathbf{N}_i \cdot \mathbf{N}_i} \tag{3.2}$$

where

$$\mathbf{F}_i = \sum_f \nabla_{\mathbf{v}_i} A_{i,f} = \sum_f \frac{1}{4A_{i,f}} \left(-\mathbf{e}_{f,1}\left|\mathbf{e}_{f,2}\right|^2 - \left|\mathbf{e}_{f,1}\right|^2 \mathbf{e}_{f,2} + \left(\mathbf{e}_{f,1} \cdot \mathbf{e}_{f,2}\right)\left(\mathbf{e}_{f,1} + \mathbf{e}_{f,2}\right)\right) \tag{3.3}$$

and

$$\mathbf{N}_i = \sum_f \nabla_{\mathbf{v}_i} V_{i,f} = \sum_f \frac{1}{3} A_{i,f} \mathbf{N}_{i,f} \tag{3.4}$$

is the average normal at the vertex. The Gaussian curvature is simply computed from the sum of the internal angles of the faces at *i*

$$k_i = 2\pi - \sum_f \theta_{i,f} \tag{3.5}$$

Elastic energy - The bending energy at each vertex for both the fluid membrane and the solid domain was computed by the built-in function *star_perp_sq_mean_curvature*,

$$E_{\mathrm{b}} = B_{\mathrm{d}} \sum_i h_i^2 \frac{A}{3} \tag{3.6}$$

where$A_i = \sum_j A_{ij}$ is the total area of adjacent facets and B_{d} is the discrete bending modulus. Note that in this built-in method, B_{d} is twice of the continuum bending modulus B, i.e., $B_{\mathrm{d}} = 2B$.

For the strain energy at each vertex over the solid domain, we used the built-in function *linear_elastic*,

$$E_{\mathrm{s}} = Y \sum_i \frac{1}{2(1+\nu)} \left(\mathrm{Tr}[C_i^2] + \frac{\nu (\mathrm{Tr}[C_i])^2}{1-\nu} \right) \tag{3.7}$$

$$C_i = \frac{1}{2}(F_i S_i^{-1} - I) \tag{3.8}$$

Where ν is the Poisson's ratio, d is the dimension, Y is the stretching modulus, and C_i is the Cauchy-Green strain tensor with unstrained Gram matrix F_i, strained Gram matrix S_i at each vertex v_i, and identity matrix I.[11] In our model, $\nu = 0.4$, and the unstrained solid domain configurations are set to be planar (extracted from the planar configuration in the initialization step 5 in Supplementary Fig. 17 above) . The total elastic energy reads

$$E_{\mathrm{elastic}} = E_{\mathrm{b}}^{\mathrm{fluid}} + E_{\mathrm{b}}^{\mathrm{solid}} + E_{\mathrm{s}}^{\mathrm{solid}} \tag{3.9}$$

Minimization protocol – Starting from the initial configuration shown in step 5 of Supplementary Fig. 17 above, Surface evolver is used to the minimize the energy while holding the solid and fluid areas at constant ratio and fixing the internal volume to achieve a given target value of $\bar{v}$.[11]

In practice minimization uses a combination of gradient descent (via the command 'g' in Surface Evolver) and Hessian step methods. In Surface evolver, there are two options to apply the Newton's method, *hessian* and *hessian_seek. hessian* directly uses the Newton's method and can potentially optimize the energy even faster, but potentially fails (leading to large mesh deformation) if the expansion is not sufficiently good. *hessian_seek* is more stable than *hessian* because it uses the Hessian matrix but it does a search along the gradient direction. In our simulation, *hessian_seek* was mainly used, with occasional trials of *hessian* for particularly sluggish minimizations. To check if an apparent minimum is a saddle point, the command *saddle* is used.

Generally, the minimization proceeds by 100 gradient descent steps followed by a Hessian minimization until the step size falls below 10^{-9}, at which point the *saddle* command is applied. This procedure is repeated at least three times.

For certain cases the final state fails to reach a minimum (it is saddle point), or to satisfy area and volume constraints. Additionally, if visual inspection of certain final states shows certain highly distorted mesh regions, or if energy differences with nearby parameter values are large, it is suspected that the configuration maybe stuck in a local minimum. For these cases, vertex averaging (for the fluid vertices only) was used to displace vertices and then the minimization procedure proceeds. If this fails, the command *jiggle* is used (for the fluid vertices only) and the minimization procedure proceeds.

Supplementary References

1. Chen, D. & Santore, M. M. Large effect of membrane tension on the fluid-solid phase transitions of two-component phosphatidylcholine vesicles. *Proc. Natl. Acad. Sci. U.S.A.* **111**, 179-184 (2014).

2. Nagle, J. F. & Tristram-Nagle, S. Structure of lipid bilayers. *Biochim. Biophys. Acta Biomembr.* **1469**, 159-195 (2000).

3. Needham, D. & Evans, E. Structure and Mechanical Propertis of Giant LIpid (DMPC) Vesicle Bilayers from 20 Degrees C below to 10 Degrees C above the Liquid Crystal Crystalline Phase Transition at 24 Degrees C. *Biochemistry* **27**, 8261-8269 (1988).

4. Schmidt, M. L., Ziani, L., Boudreau, M. & Davis, J. H. Phase equilibria in DOPC/DPPC: Conversion from gel to subgel in two component mixtures. *J. Chem. Phys.* **131**, 175103 (2009).

5. Veatch, S. L. & Keller, S. L. Miscibility phase diagrams of giant vesicles containing sphingomyelin. *Phys. Rev. Lett.* **94**, 148101 (2005).

6. Elliott, R., Katsov, K., Schick, M. & Szleifer, I. Phase separation of saturated and mono-unsaturated lipids as determined from a microscopic model. *J. Chem. Phys.* **122**, 044904 (2005).

7. Lee, S., Jeong, D. W. & Choi, M. C. Vertical order of DPPC multilayer enhanced by cholesterol-induced ripple-to-liquid ordered (LO) phase transition: Synchrotron X-ray reflectivity study. *Curr. Appl. Phys.* **17**, 392-397 (2017).

8. Soloviov, D. V., et al. Ripple Phase Behavior in Mixtures of DPPC/POPC lipids: SAXS and SANS Studies. 2nd International Workshop on SANS-YuMO User Meeting at the Start-up of Scientific Experiments on the IBR-2M Reactor; 2011 May 27-30; Joint Inst Nucl Res, Frank Lab Neutron Phys, Dubna, RUSSIA; 2011.

9. Meyer, N., Desbrun, M., Schroder, P. & Barr, A. H. Discrete differential-geometry operators for triangulated 2-manifolds. 3rd International Workshop on Visualization and Mathematics; 2002 May 22-25; Berlin, Germany; 2002. p. 35-57.

10. Brakke, K. A. Surface Evolver Manual. (2012).

11. Brakke, K. A. The Surface Evolver. *Exp. Math.* **1**, 141-165 (1992).
